# Four-Dimensional Graded Consciousness

**DOI:** 10.3389/fpsyg.2017.00420

**Published:** 2017-03-21

**Authors:** Jakub Jonkisz, Michał Wierzchoń, Marek Binder

**Affiliations:** ^1^Department of Management, Institute of Sociology, University of Bielsko-BialaBielsko-Biala, Poland; ^2^Consciousness Lab, Institute of Psychology, Jagiellonian UniversityKrakow, Poland; ^3^Psychophysiology Lab, Institute of Psychology, Jagiellonian UniversityKrakow, Poland

**Keywords:** graded consciousness, multidimensional consciousness, dimensions of consciousness, measures of consciousness, levels of consciousness

## Abstract

Both the multidimensional phenomenon and the polysemous notion of consciousness continue to prove resistant to consistent measurement and unambiguous definition. This is hardly surprising, given that there is no agreement even as regards the most fundamental issues they involve. One of the basic disagreements present in the continuing debate about consciousness pertains to its gradational nature. The general aim of this article is to show how consciousness might be graded and multidimensional at the same time. We therefore focus on the question of what it is, exactly, that is or could be graded in cases of consciousness, and how we can measure it. Ultimately, four different gradable aspects of consciousness will be described: *quality*, *abstractness*, *complexity* and *usefulness*, which belong to four different dimensions, these being understood, respectively, as *phenomenal, semantic, physiological*, and *functional.* Consequently, consciousness may be said to vary with respect to *phenomenal quality, semantic abstraction, physiological complexity*, and *functional usefulness*. It is hoped that such a four-dimensional approach will help to clarify and justify claims about the hierarchical nature of consciousness. The approach also proves explanatorily advantageous, as it enables us not only to draw attention to certain new and important differences in respect of *subjective measures of awareness* and to justify how a given creature may be ranked higher in one dimension of consciousness and lower in terms of another, but also allows for innovative explanations of a variety of well-known phenomena (amongst these, the interpretations of *blindsight* and *locked-in syndrome* will be briefly outlined here). Moreover, a 4D framework makes possible many predictions and hypotheses that may be experimentally tested (We point out a few such possibilities pertaining to interdimensional dependencies).

## Introduction

Complex as it is, the phenomenon of consciousness is hard to either measure (Seth et al., 2008; Overgaard et al., 2010; Sandberg et al., 2010; Szczepanowski et al., 2013; Wierzchoń et al., 2014) or define properly (Pareira and Ricke, 2009; Velmans, 2009; Jonkisz, 2015). It is sometimes described in rough terms as ‘all that we experience when not in a dreamless sleep or coma’ (see Searle, 2000). However, within scientific practice consciousness is mainly characterized operationally, by certain behavioral and/or neurophysiological indicators such as reportable contents, goal-directed and adaptive behavior, or *recurrent processing* in the *thalamocortical structures* (see Damasio, 1999; Crick and Koch, 2003; Edelman, 2003; Lamme, 2006; Baars, 2012; Baars et al., 2013; Feinberg and Mallatt, 2013). The reportability requirement may suggest that consciousness is limited to humans, primates and a few other highly developed species (see Carruthers, 1998), but it has also sometimes been ascribed to various other – far more ‘ancient’ – creatures (ranging from vertebrates generally to insects and lampreys – see Feinberg and Mallatt, 2013, 2016; also Edelman and Seth, 2009; Boly et al., 2013). Furthermore, its scope of application has even been extended beyond living organisms (Tononi, 2004, 2008, 2010; Chella and Manzotti, 2007; Koch, 2012; Tononi and Koch, 2014). The range of consciousness also varies with respect to biological mechanisms: that is to say, it is most often correlated with a widespread neuronal activation in higher *thalamocortical* structures (Dehaene and Changeux, 2011; Baars et al., 2013), yet more localized, lower-level activities are also sometimes deemed responsible for consciousness (see Lamme, 2006; Edelman et al., 2011; Långsjö et al., 2012). The question of just how low we should look, in searching for the most basic forms and mechanisms of consciousness, is particularly apposite when adopting, as we do here, a gradational approach to the phenomenon, as it may lead to some counterintuitive consequences, such as ascribing certain levels of consciousness to all organisms or even to primitive artificial systems (see Tononi, 2004, 2008, 2010; Koch, 2012; Jonkisz, 2015).

To be sure, it is most unlikely that individuals could be “...ordered on the basis of how conscious they are, just as they can be ordered on the basis of their age, height, or blood pressure” (Bayne et al., 2016, p. 406). Attempts to analyze consciousness in terms of successive ‘levels,’ ‘grades,’ or ‘orders’ are strongly criticized in the article just cited for being (in that author’s opinion) one-dimensional – and, indeed, we ourselves are sympathetic to the multidimensional approach presented there. Nevertheless, the fact that a given conscious creature may be ranked higher in one aspect or dimension (e.g., responsiveness, neuronal activation patterns) and lower in other (e.g., capacity or quality of content) does not render the question about the gradability of consciousness either less attractive or irrelevant (as might seem to be the case after reading Bayne et al., 2016). That question remains perfectly valid. Indeed, it is even more interesting to analyze gradability as it relates to different dimensions of consciousness – and this is, in fact, the principal aim of the present article. To be clear, the article will not discuss whether consciousness is graded or all-or-none (see Sergent and Dehaene, 2004; Overgaard et al., 2006; Windey et al., 2013, 2014; Anzulewicz et al., 2015; Andersen et al., 2016; Bayne et al., 2016), as its main objective is rather to shed light on how, and in what sense, consciousness might be graded while at the same time being multidimensional.

It is by no means clear whether gradedness pertains only to phenomenal contents themselves, or also to *global states* of consciousness, or, maybe, other aspects or dimensions of the phenomenon as well (see Bayne et al., 2016, where *global states of consciousness* are defined as “states of consciousness that characterize an organism’s overall conscious condition. An organism can be in only one global state of consciousness at a time [...],” p. 406). The article tackles that problem by seeking to determine what, exactly, it is that is, or might be, graded in consciousness. Four different gradable aspects belonging to four different dimensions of consciousness will be described; therefore, the methodological question of how to measure those hierarchies is, in effect, also in due course addressed. Furthermore, the findings presented here may ultimately help to answer certain more general questions: e.g., how science should compare and assess consciousness across different species (Griffin and Speck, 2004; Seth et al., 2005; Edelman and Seth, 2009; Boly et al., 2013; Feinberg and Mallatt, 2013, 2016), or in artificial systems (Hollande, 2003; Chella and Manzotti, 2007; Torrance et al., 2007; Clowes and Seth, 2008; Kurzweil, 2012; O’Regan, 2012), and whether it is right to say that there are developmental differences in respect of conscious grades/levels over the course of the lives of individual organisms (Stuss and Anderson, 2004; Zelazo, 2004; Kouider et al., 2013). Ultimately, it is for the future to determine whether or not these results are explanatorily advantageous; nevertheless, some of their possible applications are already outlined in the closing sections here.

## Four Dimensions of Consciousness

In order to analyze gradability as it relates to different dimensions of consciousness, it is necessary to first specify what the dimensions in question themselves consist in. Unfortunately, there is no agreement with respect to this fundamental issue, since no unambiguous characterization of the actual phenomenon exists. In spite of this, there is certainly a plethora of meanings associated with the term, and multiple varieties of consciousness that one may encounter as one engages with the different domains involved in its explanation (see Jonkisz, 2012). In fact, in the vast field of consciousness studies the phenomenon has already been described in terms that imply different dimensions: e.g., as a subjective experience or phenomenally integrated content, as a higher-order or self-representational state, as a state produced by *recurrent processing* in the *thalamocortical structures*, and as an adaptation which enables one to behave appropriately, adapt to new situations, learn, understand emotions, differentiate, and choose, etc. (Rosenthal, 1986; Baars, 1994, 2002, 2012; Block, 1995; Chalmers, 1995; Lycan, 1996; Damasio, 1999; Crick and Koch, 2003; Edelman, 2003; Tononi, 2004; Gennaro, 2005; Kriegel, 2006, 2007; Lamme, 2006; Dehaene and Changeux, 2011; Feinberg and Mallatt, 2013, etc.). Consequently, it seems reasonable to differentiate the following four dimensions of consciousness: the phenomenological, the semantic, the physiological, and the functional (adapted from Jonkisz, 2012, 2015). Each of these will be characterized below.

### The Phenomenological Dimension

The dimension refers to the *subjective* or *experiential* character of consciousness. To describe ‘what it is like’ to see something, feel pain, move, talk, think, etc., one must undergo that very experience of seeing, feeling, moving, talking, thinking, etc., oneself. This is because conscious experiences are *individuated* relative to a given subject’s perspective, and as such are unique in the way they present themselves, appear or feel (something which is in fact biologically justified – see Jonkisz, 2016). That is the simple reason for why our scientific (epistemically objective) explanations of (epistemically subjective) experience are obliged to be indirect: i.e., report-based. In short, the phenomenological dimension captures the experiential, *non-relational aspect* of consciousness.

### The Semantic Dimension

The dimension has to do with the *referential* nature of consciousness, which is sometimes also characterized as *transitive* or *intentional* (see Rosenthal, 1986; Searle, 1992, 2000). Consciousness carries a certain referential content, as it is about certain things or objects we perceive, think of, feel, remember, or imagine. Sometimes it may appear as if there is no particular object to name or point to: e.g., in cases of conscious pains, moods, emotions, etc. Yet even in these cases it may be said that consciousness refers to, or is about, the very experience of pain, mood, emotion, etc., and that the information it conveys *means something* for the subject (see Cleeremans, 2011). It may therefore be said that conscious states are *intentional* in the classical Brentanian sense, but “[b]ecause intentional states are *of* or *about* things other than themselves, for a state to have intentionality is for it to have semantic properties” (Pierre, 2003). In short, the semantic dimension captures the *relational aspect* of consciousness, not its experiential, non-relational, qualitative characteristics: we are asking here about ‘what it is about’ or ‘what its reference is,’ not ‘what it is like to have it.’

### The Physiological Dimension

The dimension concerns bodily mechanisms or *vehicles* of consciousness in a given organism. In this case our focus will be on such questions as ‘how consciousness is produced or implemented in an organism,’ and ‘what the specific processes responsible for the occurrence of a given conscious content are.’ The processes that science correlates most often with consciousness are certain types of neuronal activity: the so called *neuronal correlates of consciousness* or NCC’s (see Frith et al., 1999; Edelman and Tononi, 2000; Metzinger, 2000; Hohwy, 2009; Aru et al., 2012; Paulewicz and Wierzchoń, 2015; Koch et al., 2016). What is worth adding here is that we cannot exclude the possibility of certain non-biological, artificial systems possessing, in the future, enough causal power to produce at least primitive forms of consciousness (Chella and Manzotti, 2007; Torrance et al., 2007; Clowes and Seth, 2008; Kurzweil, 2012; O’Regan, 2012). Hence, this dimension can be generalized to include all mechanisms (be they biological or not) capable of producing consciousness, such as may be labeled *structural* or *physical*. We shall, however, leave the term ‘physiological’ in place here, as up to now consciousness has only been observed and experienced *in vivo* and *in situ*.

### The Functional Dimension

The dimension relates to the *usefulness* exhibited by conscious information: i.e., to the question of ‘what it affords’ in respect of a given creature’s actions (Gibson, 1977, 1979/1986; Engel et al., 2013). It seems that organisms able to utilize conscious information in order to, say, control, adapt or choose action patterns in given circumstances must have been more efficacious and statistically more successful – otherwise the ability to be conscious probably would not have survived (Lindahl, 1997; Baars, 2002, 2012; Griffin, 2001; Feinberg and Mallatt, 2013, 2016). Consequently, when it comes to this dimension what we are focused on is neither its experiential qualities (*what-it-is-like*), nor its reference (*aboutness*), nor the physical processes in which it is embedded (*production mechanisms*), but rather its *pragmatic function* (*usefulness in action*).

The question we shall be addressing below is, then, as follows: in what sense might consciousness be phenomenally, physiologically, semantically and functionally graded, and how might we measure such gradedness?

## Phenomenal Gradedness

Epistemically, subjective aspects of conscious seeing, touching, thinking, talking, moving, having a certain mood, feeling pain, feeling anxious, angry, etc., correspond to particular ways in which conscious contents are experienced as the processes they involve unfold. For example, when seeing something, one may experience more or less clear, vivid, blurred, bright, sharp, etc., images of certain objects or scenes in their specific situational contexts (in ways informed by both prior experiences and the actual environmental circumstances). The same goes for other types of experience, except that the list of epithets suited to describing the information available from the first-person perspective will grow. Everything that we might mention, think of, or refer to in response to the questions ‘What is it like to have that particular conscious content?’ and ‘How was that particular content experienced?’ relates to the phenomenal dimension. Any other party, except the experiencing subject, is bereft of such an exact and direct ‘view from within,’ and therefore must rely upon the testimony of the subject regarding what has been experienced, together with observations of his or her behavior.

In what sense, then, might consciousness be phenomenally graded, and how could we measure this? Experiences are more or less vivid, sharp, intense, clear, rich, detailed, etc. It may be said, therefore, that what is graded is their vividness, sharpness, intensity, clarity, etc. – or, in more general terms, their *informational quality.* Several experimental procedures have been proposed in psychology to tackle this problem: e.g., signal-detection-based methods, free verbal reports, structured verbal report scales, and no-report measures or phenomenological methods, to name just a few (for a review of these, see, for example: Heavey and Hurlburt, 2008; Overgaard and Sandberg, 2012; Wierzchoń et al., 2012; Overgaard, 2015; Tsuchiya et al., 2015). Here, we shall focus on structured verbal report scales: i.e., on so called *subjective measures* of consciousness, which target the phenomenal dimension of experience and aim to estimate in quantitative terms the graded informational quality of certain contents of consciousness.

A prominent and oft-cited example is the *Perceptual Awareness Scale* (PAS), which estimates the subjective visibility of a stimulus on a four-point verbal scale whose response categories are labeled (1) “no experience,” (2) “brief glimpse,” (3) “almost clear experience,” and (4) “clear experience” (see Ramsøy and Overgaard, 2004). Most of the papers using these methods show that the informational quality captured with this scale is graded, in the sense that (1) participants tend to use all available responses, (2) a correlation with accuracy is observed across all four points of the scale, and (3) the psychophysical functions of response accuracy and stimulus visibility change gradually as a function of stimulus salience (manipulated in terms of presentation time or stimulus contrast – see, for example, Windey and Cleeremans, 2015). Similar analyses have been proposed for other structured verbal report scales, such as a *confidence ratings* scale or a *post-decision wagering scale* (Sandberg et al., 2010, 2011).

When we rely on subjective reports, as the above-mentioned methods do, what is it that we are actually measuring? Are we directly measuring the quality of conscious experience, or the quality of information about these? Put another way, do the reports given by participants capture the variability of the phenomenal aspect of consciousness, or rather reflect their introspective or metacognitive ability (Overgaard and Sandberg, 2012)? The next section should hopefully shed some light on this issue. It will also enable us to show the differences across the various measures, as those are, at least partially, a matter of a semantic gradation. It is also worth adding here that not every creature is able to report its own experiences, which of course does not mean that it has no experiences of a certain quality! What it does mean, however, is that report-based, *subjective measures* are going to be blind to that.

## Semantic Gradedness

Conscious contents are not just experienced in some particular way or other: they are also about something or other. In other terms, besides the subject of consciousness, there is also an object it refers to. “The subjective (subject-related) and intentional (object-directed) dimensions of experience are intertwined with each other as dimensions correlatively constituting a single act of consciousness” (Legrand, 2007, p. 577). There is a variety of *referential objects* that a given subject may be aware of: e.g., certain perceptions, emotions, feelings, thoughts, memories, etc. In asking whether consciousness is semantically graded, we are really asking about the gradability of the relation between some subjectively experienced content and its reference. The relation may be graded in a sense of being more or less abstract. For example, we may not only consciously see something (lower-order visual awareness), but may also be aware of seeing (higher-order awareness); in such cases, the awareness of visual objects is less abstract than the awareness of the process of seeing (see, in this regard, the many examples of this kind presented in Dretske, 2006). More generally, if a subject may not only be ‘conscious of something,’ but also ‘aware of being conscious of something,’ or even ‘conscious of the fact that he or she was aware of being conscious of something,’ etc. (see Jonkisz, 2012), then it may be said that consciousness is semantically graded (with *abstractness* as a gradable aspect).

This relatively straightforward idea of contents of consciousness being reflected in higher-order states may be traced back as far as Aristotle and his theory of *inner sense* (see *De Anima*, 425b, pp. 12–25, in Hamlyn, 1968, pp. 47–48). It is also visible in certain models of consciousness known today: for example, in so called *higher-order theories* (see Rosenthal, 1986; Lycan, 1996; Gennaro, 2005). The idea of *metacognition* may also be interpreted partly in these terms (see Overgaard and Sandberg, 2012; Shea et al., 2014). But, we may ask, is the phenomenon of consciousness actually graded semantically in such a simple, linear fashion? The issue certainly needs to be explored in greater detail, as it is not clear, for example, how we should interpret cases of perception of higher- *versus* lower-level (or lower-order) features, such as meanings or object categories, as distinct from colors, motions, locations, etc. [This scenario is described, for instance, by Bayne et al. (2016, p. 409), when relating *global states* to contents, and by Windey et al. (2013, 2014) in their experimental tasks involving different *levels of processing*]. Is the semantic relation in such cases more or less abstract because the referential objects themselves are more or less abstract, or because a given subject needs higher, metacognitive orders of awareness to extract or refer to the higher-order features (categories, meanings, etc.)?

Unfortunately, here also (as in the case of qualitative, phenomenal aspects) we do not observe more or less abstract ‘meanings,’ or their ‘referential objects,’ directly, either in the brain or in the behavior of a given participant. Yet, when the latter behaves in a certain way, reports knowing certain things, and/or his or her brain reveals specific activity patterns, it is possible to infer with varying degrees of probability what he or she is aware of, and it may then also be feasible to assess just how abstract that awareness could be. For example, when a given person is asked to watch people playing basketball and let us know when a gorilla has been spotted (as in the classic experiment testing *inattentional blindness* – see Simons and Chabris, 1999), and the participant fulfills this complex task accurately, we may infer that he or she has not only consciously seen something, but must also have been aware of what it was – and, in order to let us know, must even have been aware of having been aware. However, if we wish to assess or, more importantly, to measure semantic orders of awareness, we will probably need to design specific order-related tasks.

Differentiation in terms of semantic orders might prove useful when it comes to indicating the differences between the *subjective measures* mentioned in the previous section. For example, *PAS* and *Confidence Ratings* methods are used in typical *backward masking* scenarios: i.e., when participants are presented with a near-threshold stimulus followed by a mask, and then judge the stimulus according to both objective (e.g., identification) and subjective (e.g., visibility) measures (see, for example, Sandberg et al., 2010, 2011; Wierzchoń et al., 2014). Both these methods are based on structured subjective reports, yet to fulfill the task requirements, participants have to report specific information they are aware of which actually seems to differ as regards the order of abstraction involved. With the *PAS*, a participant has to be aware of the quality of his or her own experience of an object flashed on the screen (e.g., a face), whereas in the case of the *Confidence Ratings* a participant has to be aware of his or her own confidence in his or her visual experience of that object (see Wierzchoń et al., 2014). It seems, therefore, that *PAS* methods require a semantically lower-order form of awareness than do *Confidence Ratings* methods. On the other hand, assuming that first-order awareness corresponds to awareness of a stimulus (e.g., a face), and not to awareness either of the experiential quality of the stimulus or of one’s confidence in one’s own awareness of the stimulus, it may be said that neither the *PAS* nor the *Confidence Ratings* approach measure the awareness of a stimulus directly. Even the *PAS* inevitably requires a metacognitive judgment about stimulus visibility (i.e., whether I indeed saw a face, and how I should categorize the quality of that experience in the light of the *PAS* categories). Summarizing these considerations, it may be stated that at the lowest-order level there is ‘awareness of the stimulus’ (e.g., a face seen on the screen), that above this there is then a more abstract ‘awareness of the visibility of the stimulus’ (as required by the *PAS*), and that beyond this there is the most abstract ‘awareness of confidence about the visibility of the stimulus’ (as required by the *Confidence Ratings* approach).

## Physiological Gradedness

Consciousness, as observed in both humans and certain animals, is correlated with the presence of certain physiological processes: i.e., specific forms of metabolic and electrochemical activity observed in the nervous systems of conscious organisms. Using lesion studies (Laureys et al., 2015), modern *neuroimaging* methods (see Bandettini, 2009), *electroencephalography* (EEG) and *transcranial magnetic stimulation* (TMS), we can observe and modulate the way these processes unfold in the human or animal brain over both very short and longer periods of time (see Edelman and Seth, 2009; Bisenius et al., 2015; Koch et al., 2016). It is thus possible to establish not only their location, but also, more importantly, their specific *activity patterns.* Although there are lots of knowledge-gaps and disagreements about the *neuronal correlates of consciousness* (see, for example: Crick and Koch, 1990; Hohwy, 2009), it may be said that in humans the phenomenon is most probably caused or generated within the *thalamocortical* system and the ascending activating systems originating in the *brainstem* and *subcortical* regions (see Blumenfeld, 2016, pp. 3–29). In what sense, then, may consciousness be physiologically graded, and how can we measure and model it?

The most appropriate approach here would surely be to attempt to define the neural underpinnings of consciousness as some sort of general property of overall dynamic brain activity patterns. One of the most developed proposals in this regard is the theory of *integrated information* of Tononi (2004, 2008). *Integration* is defined as the effective sharing of information between parts of the system (Tononi, 2008, 2010; see also Tononi et al., 2016, for an updated version of the theory). Effective information associated with the “weakest link” of some possible system bipartition can be represented by a single numerical value named Φ, which represents the whole system’s capacity to integrate information. Another formulation of the integration idea is Anil Seth’s notion of *causal density* (Seth et al., 2006). This measure aims at capturing dynamic heterogeneity among network elements and their global dynamic interaction. As in the case of Tononi’s proposal, causal density can be computed as a single value “cd,” which can also change gradually, its values depending on the fraction of interactions between neuronal elements that are causally significant at a given moment (Seth et al., 2006; Seth, 2008; Barrett and Seth, 2011).

Recently, Massimini and colleagues have proposed an empirical indicator of the capacity of the brain to generate complex patterns of activity: *a perturbational complexity index* (PCI) (Casali et al., 2013). This parameter is based on measuring the cortical patterns of electrophysiological responses to neural network perturbation induced by repeated TMS pulses. In short, PCI, using algorithmic complexity calculations, is assumed to measure the capacity of the thalamocortical system for effective and rapid interactions. According to its proponents, PCI should be interpreted as an EEG-based indicator of integration and differentiation of neural activity, and thus also directly relates to the basic tenets of Tononi’s theory. Extensive studies on normal subjects in wakefulness, at various stages of sleep, and undergoing various levels of anesthesia, as well as post-coma patients with severe brain damage, have proved that the single value of the PCI indicator can indeed distinguish between various forms of impairment of consciousness (Casali et al., 2013). To be specific, higher values of the PCI indicate normal waking consciousness, while lower levels point to forms of absent or severely impaired consciousness. In a follow-up study, with numerous subject samples (Casarotto et al., 2016), the predictive accuracy of PCI was tentatively confirmed by observing relatively high values of PCI (within the range of conscious controls) in a group of patients who had been behaviorally diagnosed as vegetative, thus implying a capacity to retain consciousness despite a limited capacity for motor response.

It may be said that all the above-mentioned proposals “attempt to quantify the balance between integration and differentiation exhibited by a neural system” (Seth et al., 2006, p. 10800) and aim at measuring and modeling (in their own way) the overall *dynamic complexity* of the activity patterns correlated with consciousness. Although, the *complexity* is understood differently in these approaches (either as a global feature of the interactions between relevant neural networks or as a feature of cortical EEG-responses to TMS perturbations), it might qualify as a reasonable candidate for a gradable element in the physiological dimension. Unfortunately, although a certain level of neural complexity seems necessary, it still remains uncertain whether high complexity-values will possess enough predictive accuracy to indicate consciousness consistently.

## Functional Gradedness

It seems intuitively right to assert that consciousness plays a vital role in many forms of complex behavior: in decision making, action planning, problem solving, thinking, reasoning, learning, etc. (see Baars, 2002, 2012; Merker, 2005; Morsella, 2005; Cohen and Dennett, 2011). At the same time, it also appears closely related to various cognitive processes or functions, such as attention, language, and working memory (see Baars and Franklin, 2003; Lamme, 2006; Bor and Seth, 2012). A number of studies – that together make up the so called ‘integration consensus’ (a term coined in Seth, 2009) – suggest that the role of consciousness is to integrate signals and information from different internal systems (e.g., memory, motor, sensory) and external resources (see Baars, 1994, 2002; Dehaene et al., 1998; Edelman and Tononi, 2000; Dehaene and Naccache, 2001; Edelman, 2003; Tononi, 2004, 2008, 2010; Seth et al., 2005; Seth, 2009; Dehaene and Changeux, 2011; Edelman et al., 2011; Palmer and Ramsey, 2012; Baars et al., 2013; Tononi and Koch, 2014). There are, however, examples of unconscious decision-making (Soon et al., 2008), *implicit learning* (Stadler and Frensch, 1997), attention without consciousness (Koch and Tsuchiya, 2007), unconscious working memory (Soto et al., 2011), and neuronal integration without consciousness (Mudrik et al., 2014). So, is it possible for unconscious processes to perform all these functions? Unfortunately, it does not, at least for the moment, seem feasible to give any definite answer to this (see Hassin, 2013; Hesselmann and Moors, 2015).

All the same, the assertion that the capacity for consciousness holds rather high evolutionary value (Lindahl, 1997; Griffin, 2001; Feinberg and Mallatt, 2013, 2016) simply by virtue of being *useful* or *efficacious* in certain conditions still seems justified. That view is by no means a new one, as it was already put forward by William James: “[t]he particulars of the distribution of consciousness, so far as we know them, point to its being efficacious… it seems an organ, superadded to other organs which maintain the animal in the struggle for existence; and the presumption of course is that it helps him in some way in the struggle…” (James, 1890, cited in Seth, 2009). Of course, unconscious (or less conscious) information processing is also useful, and even more efficacious than conscious one, in many situations (e.g., when speed is more crucial than accuracy, when suitable action patterns are well-known or routinely performed, etc.). Therefore, the crucial functional question does not concern the adaptive value of consciousness, which seems obvious, but rather the reason why it is valuable (in the sense of being useful) in certain situations, or what it affords its possessor. As far as we know, consciousness enables *flexible behavior*, which is revealed in a creature’s ability to alter and adapt its actions in line with developing changes in its environment, to correct motor, perceptual or cognitive errors, to compare predictions with actual conditions, to detect differences, to test and sample planned action virtually, etc. (see Pally, 2005; Seth, 2009; Baars et al., 2013). It seems quite likely that such *flexibility* in acting may be the reason why consciousness is most efficacious in certain circumstances. If that is so, then indeed, the *flexibility* which consciousness offers may be proposed as a reasonable candidate for its distinctive function.

So, in what sense might consciousness be functionally graded? The usefulness of the flexibility in acting made possible by conscious processing of this or that information depends on the specific conditions of the moment. Therefore, when conditions change, the same information may turn out to be more or less useful when conscious – and, conversely, in the same conditions, different information may present varying degrees of usefulness (in virtue of enabling flexibility in respect of a different set of actions). Ultimately, then, the functional gradedness of consciousness may be identified with *varying degrees of usefulness* offered by sets of flexible actions enabled by conscious processing.

How might we measure the degrees of usefulness enabled by conscious processing? This definitely does not seem like a straightforward task, as it is structured and limited by both objective and subjective factors. Objective factors pertain to ongoing conditions in a given creature’s surroundings (spatio-temporal relations, exposure times, reaction times, locations, motions, etc.). Subjective factors, on the other hand, are concerned with everything that is subject-dependent: actual physiological states, repertoires of action patterns, preferences, needs, expectations, etc. All these are unique to a given creature-subject, and will have been shaped by the latter’s individual history, by genetic and epigenetic possibilities and traits, and by actual environmental interactions (see Jonkisz, 2016). At least some of these subjective factors will not be directly accessible (preferences, moods, expectations, feelings, etc.), so the measure of functional gradedness, if developed, will have to be at least partly indirect (i.e., based on reports or probability calculations).

The functional gradedness of consciousness, understood in terms of *varying degrees of usefulness*, fits well with the increasingly popular *Bayesian brain* metaphor, according to which our cognitive system is a kind of *prediction* or *inference machine* (an idea already introduced into neuroscience by Helmholtz – see Helmholtz, 1866/1962). The information with the highest level of *expected efficacy* may be interpreted as being (in the Bayesian sense) statistically the most useful if conscious (see Jonkisz, 2016). If neuronal systems indeed work according to the principles of Bayesian statistics, then the most functional information (from the predictive perspective of some given system) may be identified with consciousness. Yet it remains debatable how the nervous system achieves this: i.e., whether it actually calculates the probabilities or rather just *samples* expected efficacy (see Sanborn and Chater, 2016; Seth and Friston, 2016).

## Discussion

To determine whether it is the phenomenal, semantic, physiological or functional hierarchy that is under consideration (especially where this cannot be directly inferred from the context), certain more precise conceptual demarcations between such terms as ‘grades,’ ‘orders,’ ‘levels,’ and ‘degrees’ of consciousness may well prove useful (however, the terms are sometimes used interchangeably; see, for example, Bayne et al., 2016). If that is indeed the case, the following practical guidelines should perhaps be embraced. Firstly, the expression ‘grades of consciousness,’ often used in the literature in relation to subjective measures, is best suited to expressing the idea of phenomenal quality grades. Meanwhile, the semantic-abstraction hierarchy is best captured by the notion of ‘orders of consciousness’ (as in cases of higher-order theories). On the other hand, as regards the physiological dimension and the idea of progressive complexity revealed by neuronal activity patterns, the term ‘levels of consciousness’ is most appropriate. Finally, when it comes to the hierarchy of functional usefulness, the term ‘degrees of consciousness’ seems suitable.

At this point it is also worth echoing the caveat issued by Bayne et al. (2016), to the effect that the “science of consciousness has been overly hasty in employing the notion of a conscious level as a central theoretical construct.” We ourselves are not convinced, either that the notion “has become [indeed] a key theoretical construct in the science of consciousness,” or that it is understood one-dimensionally in most instances of its application. The notion is employed by clinicians with reference to a patient’s overall conscious state, the latter being assessed (mainly quantitatively) according to various protocols and scales (e.g., *Glasgow Coma Scale*, *Coma Recovery Scale* – see Schnakers et al., 2008; see also Giacino, 2005). Such diagnoses, crucial for recovery prognoses in post-comatose patients with disorders of consciousness, have to be simple: hence their linear form. Yet the data used in the assessment protocols actually pertains to many of the dimensions of consciousness described above. For example, the patient’s behavioral responsiveness is a functional parameter, while the extent and localisation of brain lesions relates to the physiological dimension. Patients are also asked certain questions (“Is your name Donald?”), or may be given certain instructions (“Look up!,” “Touch the table!,” “Show us how to use a fork!,” or even something more abstract, such as “Imagine playing tennis!” – see Owen et al., 2006). To complete such tasks, patients must undergo a specific conscious experience with a definite referential content; hence, the phenomenological and semantic dimensions are also involved. If that is so, then the one-dimensional character of levels of consciousness amounts to a mis-specification: scales may yield single scores implying some sort of linear continuum of conscious levels, but in fact they are using multidimensional data. We do not think, therefore, that there is a justified worry either about using the term ‘level of consciousness’ in ways other than those with specifically clinical connotations, or about such a practice directly engendering a one-dimensional account of consciousness.

Is it useful, either theoretically or explanatorily, we may ask, to dissect consciousness into four distinct dimensions and gradable aspects? In fact, the dimensions have already proved useful, enabling us to link together the numerous varieties of consciousness and fit them all into a fourfold taxonomy (see Jonkisz, 2012, 2015). Moreover, it has been shown here that the semantic orders of consciousness also prove helpful when seeking to characterize new and important differences between *subjective measures of awareness* (see Wierzchoń et al., 2014). It is hoped, moreover, that the conception of consciousness as graded in four different dimensions (or as ‘4D’) outlined here will also prove explanatorily useful in the interpretation of a variety of phenomena. For example, *locked-in syndrome* may be interpreted within the 4D framework as a case where a patient is able to present high grades of phenomenal quality, abstract semantic orders, and complex physiological levels, but lowered degrees of functional usefulness of conscious processing. That is because his or her consciousness is functionally inefficacious in respect of motor actions, yet may still be functionally intact when it comes to reasoning, thinking, etc. The *blindsight* phenomenon, on the other hand, may be described as a condition in which *visual consciousness* presents very low phenomenal grades and semantic orders, as a blindsighted patient, in most cases, has barely any experiences or referential contents that he or she is able to report. Also, levels of physiological complexity of the visual process will most likely be lowered – though this depends on the extent of lesions in the visual areas (see Stoerig and Barth, 2001; Overgaard et al., 2008). Nevertheless, in cases of *blindsight* awareness may still possess a certain degree of functionality, as the information may prove efficacious up to a certain point where spatial navigation is concerned, or when guessing what has been seen (see Mazzi et al., 2016). Consciousness graded in four dimensions, moreover, presents us with a potentially highly advantageous scenario in respect of descriptions and comparisons concerning consciousness in non-human animals, in humans in early infancy, and in artificial-systems. (At least, we may hypothesize that this is so: for example, a child’s consciousness will naturally be less semantically abstract, but a young infant is likely to have phenomenally more distinct and intense sensory experiences in each modality, as their sensory areas will be less integrated within the brain.) It seems reasonable to assert that consciousness, as something manifested across different species, at different developmental stages, or in different categories of system, is unlikely to be successfully described and compared if these four gradable aspects are not taken into account.

It is also hoped that the 4D framework will ultimately result in many testable hypotheses and predictions being generated. Such possibilities will mostly concern possible dependencies between the four hierarchies. For example, in the realm of the dimensions proposed it would be interesting to determine how phenomenal quality is related to functional usefulness (e.g., whether low-quality grades diminish the degree of usefulness), or to investigate the extent to which the parameter of physiological complexity affects orders of abstraction (It can be assumed here that higher metacognitive orders of abstraction will require a high level of complexity). It also seems worthwhile to explore whether higher semantic orders could ever correlate with low phenomenal quality. In the latter case, if semantically abstract information were to result predominantly in high phenomenal quality, while lower-order information correlated with a more unstable quality, then such results would be compatible with the *Level of Processing Hypothesis* (see Windey et al., 2013, 2014), which claims that low-level stimuli result in more gradual experience. This would then compromise both the all-or-none approach proposed by Sergent and Dehaene (2004, where relatively abstract stimuli [word number] resulted predominantly in either very high or very low quality) and the graded-consciousness account described by Overgaard et al. (2006, where relatively less abstract stimuli [orthogonal lines] resulted in much more unstable quality).

## Conclusion

Our major aim in this article has been to answer the following question: what, exactly, is it that is graded in the case of consciousness? Our proposal has been that there are four different gradable aspects of conscious information: *quality*, *abstractness*, *complexity* and *usefulness*, which belong to four different dimensions, these being understood, respectively, as *phenomenal, semantic, physiological*, and *functional*. Consequently, conscious information processing in a 4D-framework may be said to present four different hierarchies: *grades of phenomenal quality, orders of semantic abstraction, levels of physiological complexity*, and *degrees of functional usefulness*. The approach set out here not only enables us to draw attention to certain new and important differences in respect of *subjective measures of awareness*, but also allows for an innovative interpretation of a variety of well-known phenomena (Amongst these, the interpretations of *blindsight* and *locked-in syndrome* entailed have been briefly outlined above). Moreover, it makes possible many predictions and hypotheses that can be experimentally tested – a few of which were briefly described in the previous section. Hopefully, the results will ultimately help to explain, or even conclusively answer, some of the problems described in the introduction. Of course, to determine whether the direction taken by this inquiry is the right one, still more research is needed, and this may ultimately lead to the development of forms of measurement oriented specifically toward this or that particular gradable aspect of consciousness. Above all, it is hoped that the 4D framework will enable discussions about the hierarchical nature of consciousness to be pursued with significantly greater accuracy and clarity in the future.

## Author Contributions

JJ conceived the initial ideas and wrote the manuscript, however all authors listed, have made substantial contribution to the work, and approved it for publication.

## Conflict of Interest Statement

The authors declare that the research was conducted in the absence of any commercial or financial relationships that could be construed as a potential conflict of interest.
